# Effect of the preparation of lime putties on their properties

**DOI:** 10.1038/s41598-017-17527-3

**Published:** 2017-12-08

**Authors:** Eva Navrátilová, Eva Tihlaříková, Vilém Neděla, Pavla Rovnaníková, Jaroslav Pavlík

**Affiliations:** 1 The Czech Academy of Science, Institute of Scientific Instruments, Brno, 612 64 Czech Republic; 2 Institute of Chemistry, Faculty of Civil Engineering, Brno University of Technology, Brno, 602 00 Czech Republic; 3 Lhoist, Lime kiln Čertovy schody a.s., Tmaň, 267 21 Czech Republic

## Abstract

In the study of lime as the basic component of historical mortars and plasters, four lime putties prepared from various kinds of lime of various granulometry and by various ways of preparation were evaluated. The rheological properties and micro-morphologic changes, growing of calcite crystals, and rate of carbonation were monitored. The lime putty prepared from lump lime achieves the best rheological properties, yield stress 214.7 Pa and plastic viscosity 2.6 Pa·s. The suitability of this lime putty was checked by testing the development of calcium hydroxide and calcite crystals using scanning electron microscopy and environmental scanning electron microscopy. The disordered crystals of calcium hydroxide exhibit better carbonation resulting in the large crystals of calcite; therefore, the mortar prepared from the lump lime has the highest flexural strength and compressive strength 0.8/2.5 MPa, its carbonation is the fastest and exhibits the longest durability. Also, thanks to the micro-morphological characterization of samples in their native state by means of environmental scanning electron microscopy, the new way of lime putty preparation by mixing was proven. The preparation consists in the mechanical crash of the lime particles immediately after hydration. This enables the properties of putty prepared from lump lime to be nearly reached.

## Introduction

In the past lime putty was traditional material with a wide application in construction. It used to be prepared from lump lime and it featured good workability, plasticity and durability. The traditional preparation of lime putty was replaced by modern chemical-technological processes. Lime putty is essential for the restoration of historic plaster, wall painting, sgraffito etc^1^. Lime putty is obtained by the hydration of calcined limestone in an excess of water according to the following equation:

1 $${\rm{CaO}}+{{\rm{H}}}_{2}{\rm{O}}\to {\rm{Ca}}{({\rm{OH}})}_{2}+65\,{\rm{kJ}}\cdot {{\rm{mol}}}^{-1}$$

It is a strongly exothermic reaction which leads to the enlargement of volume. The ratio of change in the particles’ volume of CaO to Ca(OH)_2_ is 16.9/33.2. Lime putty is an aqueous suspension of calcium hydroxide particles. A small proportion of calcium carbonate can be present due to incomplete calcination of limestone or reaction with carbon dioxide. The process of hydration of CaO is dependent upon the reactivity of quicklime, which is influenced by the temperature of calcination of CaCO_3_. CaO is released from limestone and the pore structure of grains is generated during calcination. The physical properties of quicklime, such as porosity, density, grain size and hydration rate, are dependent on the temperature of calcination. Furthermore, the process of CaO hydration is dependent on the distribution of particle size, the quantity and quality of the added water, the temperature of the water, the presence of impurities in the quicklime, and the presence of ions in the mixing water^2,3^. The specific surface area is the most reliable parameter which can be used to evaluate the reactivity of quicklime. The higher specific surface area of quicklime is the reason for its higher reactivity^4–7^. The burning of CaO leads to changes in the microstructure and these differences in the microstructure of CaO crystals burnt at different temperatures result in different hydration activity of CaO^8^. The hydration process of quicklime is also affected by the particle size of CaO. The finer particles are more sintered and thus they cause a low reactivity of quicklime. The coarser particles will contain some amount of unburned CaCO_3_, which is an inert material in quicklime^4,9^. Various methods and the temperature of hydration of CaO lead to obtaining lime putty with a different morphology, size, and size distribution of calcium hydroxide crystals. The calcium hydroxide crystals with a size of approximately 100 –110 nm originate during the hydration in the excess of water and at a lower temperature^5^. Calcium hydroxide hydrated in the excess of water exhibits crystals which have clearly visible edges. Calcium hydroxide hydrated in a smaller amount of water is without the sharp edges of crystal and tends to form agglomerates^10^.

The long-term maturing of lime putty in an excess of water has a beneficial effect on its quality. Such stored lime putty has better plasticity, workability, and it retains sufficient water to prepare plaster of a high durability^11–13^. The microstructure of calcium hydroxide is changed during the maturing of lime putty^14,15^ and the Ca(OH)_2_ crystals diminish. The Ca(OH)_2_ crystals with a size of between 37–50 nm occur in freshly hydrated lime - the crystals have a size of 34 nm at the age of two months and 28 nm at the age of 14 months. The specific surface area of the calcium hydroxide increases with this reduction in the size of the calcium hydroxide crystals^11,14–16^. Furthermore, the calcium hydroxide crystals change their form from large prismatic crystals to submicrometre plate crystals and secondary nucleation of nanometre plate crystals^11,15,17^. The change in the original prismatic crystals into smaller submicrometre plate crystals with a higher specific surface area leads to an increased adsorption of water and thereby increases plasticity and workability^10,11,15,17^. Atzeni *et al*.^16^ examined the effect of the maturation of lime putty on its viscosity. They prepared three lime putties with different times of maturation. Observation of the lime putties by TEM revealed that the calcium hydroxide crystals reduce in size over time. The oldest lime putty showed the greatest shear resistance and it was even more viscous than the three-month-old lime putty as well as the non-matured lime putty. The non-matured lime putty contains the agglomerates of calcium hydroxide and these agglomerates are covered by a thin film of water, which acts as a lubricant, and so this lime putty is extremely fluid. The prolonged ageing of lime putty leads to a higher viscosity, which is caused by the change in its microstructure and the specific surface area of calcium hydroxide during the ageing process. Analogous to the investigation of lime, Paiva *et al*.^18^ prepared lime mortars with a different maturation period. The mortars matured for seven days were more viscous in their fresh state, showed higher strengths at the age of 90 days, and exhibited a lower capillary absorption coefficient than the mortars without maturation. Margalha *et al*.^19^ found that slaked lump lime provides a binder with a higher proportion of active lime than powdered lime. The mortars prepared from the micronized lime show a higher porosity with larger pores, leading to a reduction in their mechanical strengths. The mortars prepared from lump lime exhibit higher strengths, but they require over-ageing treatment because of their shrinking.

## Results

### Rheology of the lime putties

**Figure 1:**
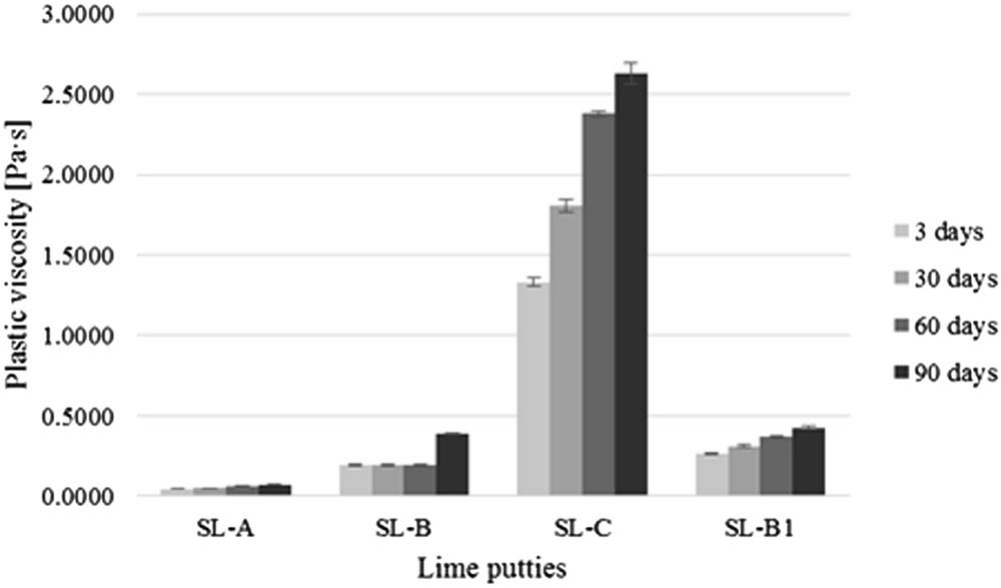
Average values of plastic viscosity of lime putties at different ages.

**Figure 2:**
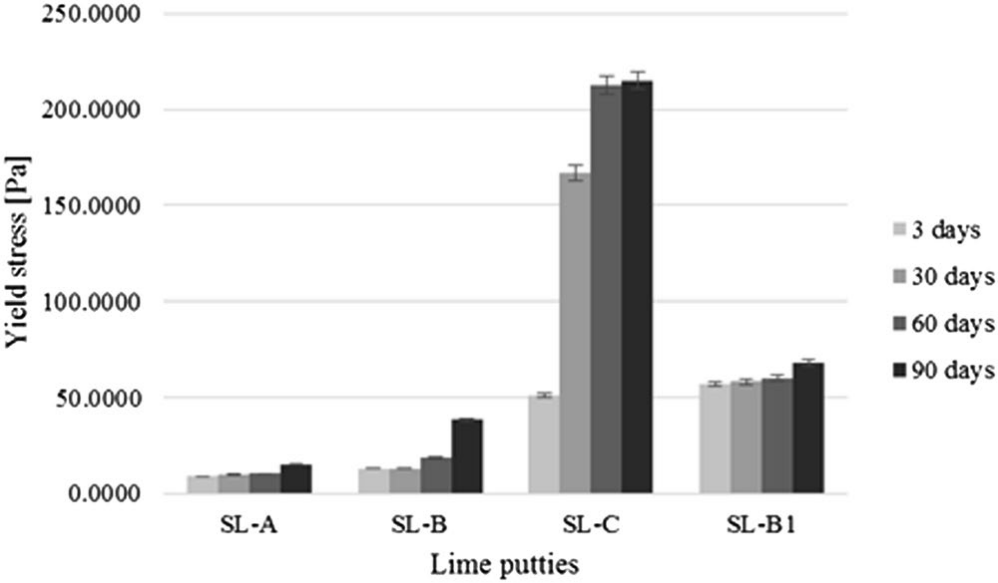
Average values of yield stress of lime putties at different ages.

The results of the measurement of the plastic viscosity and the yield stress of lime putties SL-A, SL-B, SL-C, and SL-B1 during their maturation at the age of 3, 30, 60, and 90 days are shown in Figs 1 and 2. The graphs show an increase in the plastic viscosity and the yield stress of the lime putties with a prolonged period of maturation. The increase in the values of the plastic viscosity and the yield stress in lime putties SL-A, SL-B, and SL-B1 is not so apparent at the ages of 3, 30, and 60 days compared to lime putty SL-C. The values of the plastic viscosity and the yield stress of lime putty SL-B increase significantly at the age of 90 days. The increase in the values of the plastic viscosity and the yield stress is slow at the age of 90 days for lime putties SL-A and SL-B1. The highest plastic viscosity and yield stress were found in the sample from lump lime C. Lime putty SL-A, prepared from lime containing grains under 90 µm, showed the lowest plastic viscosity and yield stress. Lime putty SL-B, prepared from lime containing grains under 200 µm, reached only slightly higher values of plastic viscosity and yield stress than lime putty SL-A. However, the differences in the values of yield stress and plastic viscosity were greater at the age of 90 days for lime putties SL-A and SL-B. The disruption of the particles by mixing affected the plastic viscosity and yield stress of lime putty SL-B1, which reached higher values of plastic viscosity and yield stress than lime putty SL-B, which was not exposed to mixing after hydration, and the grains of the lime are not in any way disrupted. The results of the rheological measurements are consistent with previously published results^16,18,19^.

### Image of the microstructure of the lime putties

**Figure 3:**
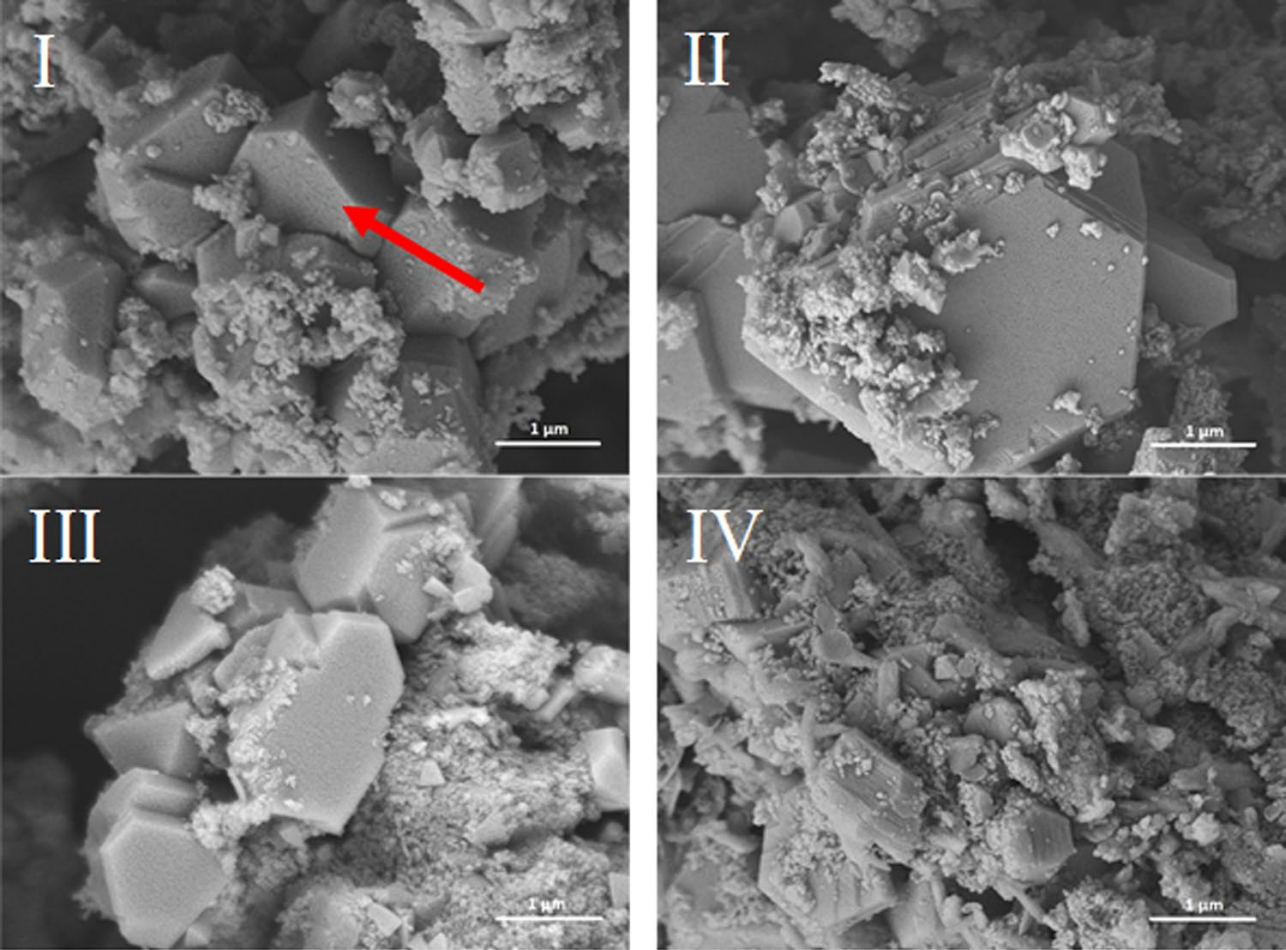
Lime putty SL-A at the age of 3 days (**I**), 30 days (**II**), 60 days (**III**) and 90 days (**IV**).

**Figure 4:**
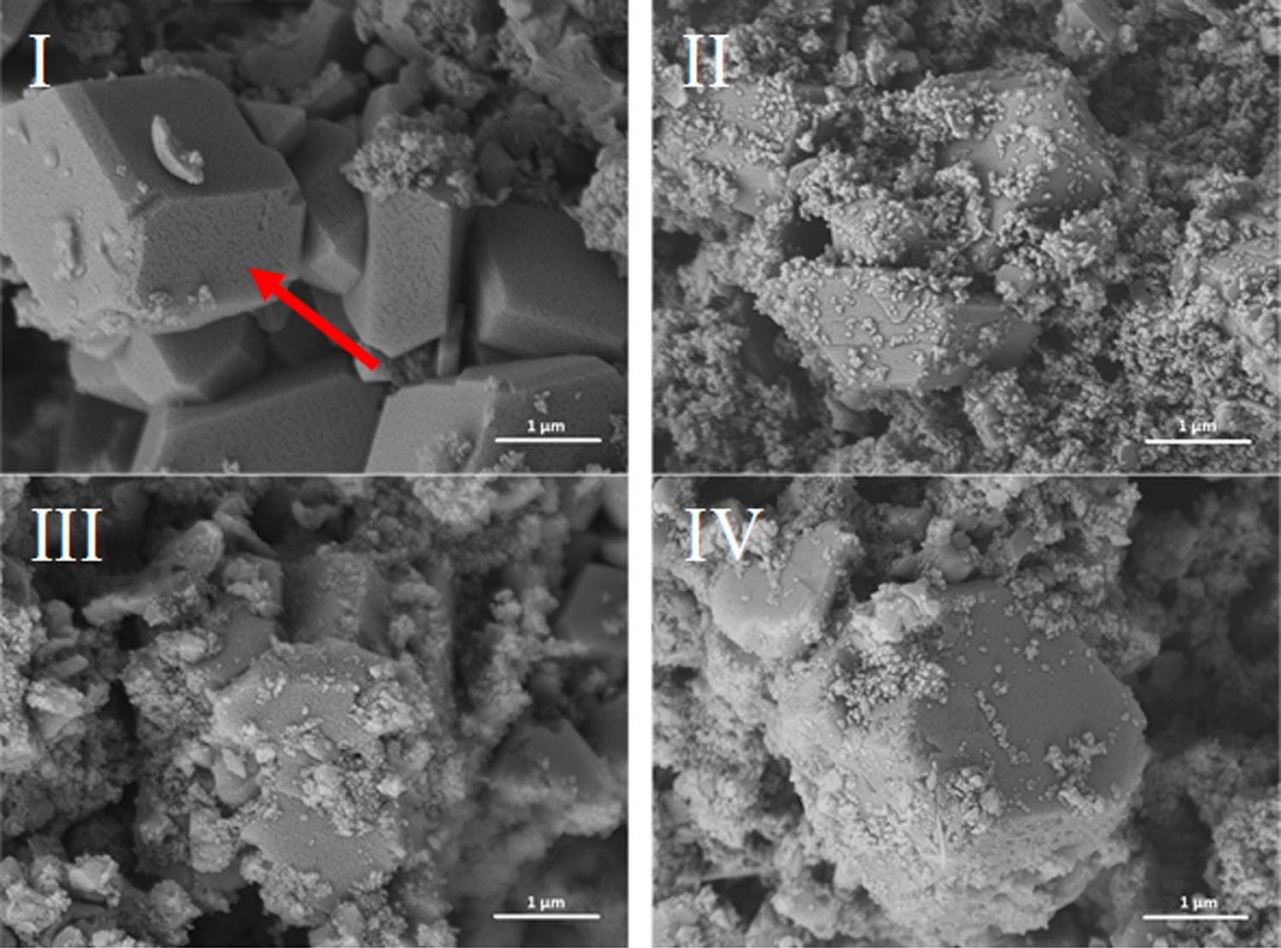
Lime putty SL-B at the age of 3 days (**I**), 30 days (**II**), 60 days (**III**) and 90 days (**IV**).

**Figure 5:**
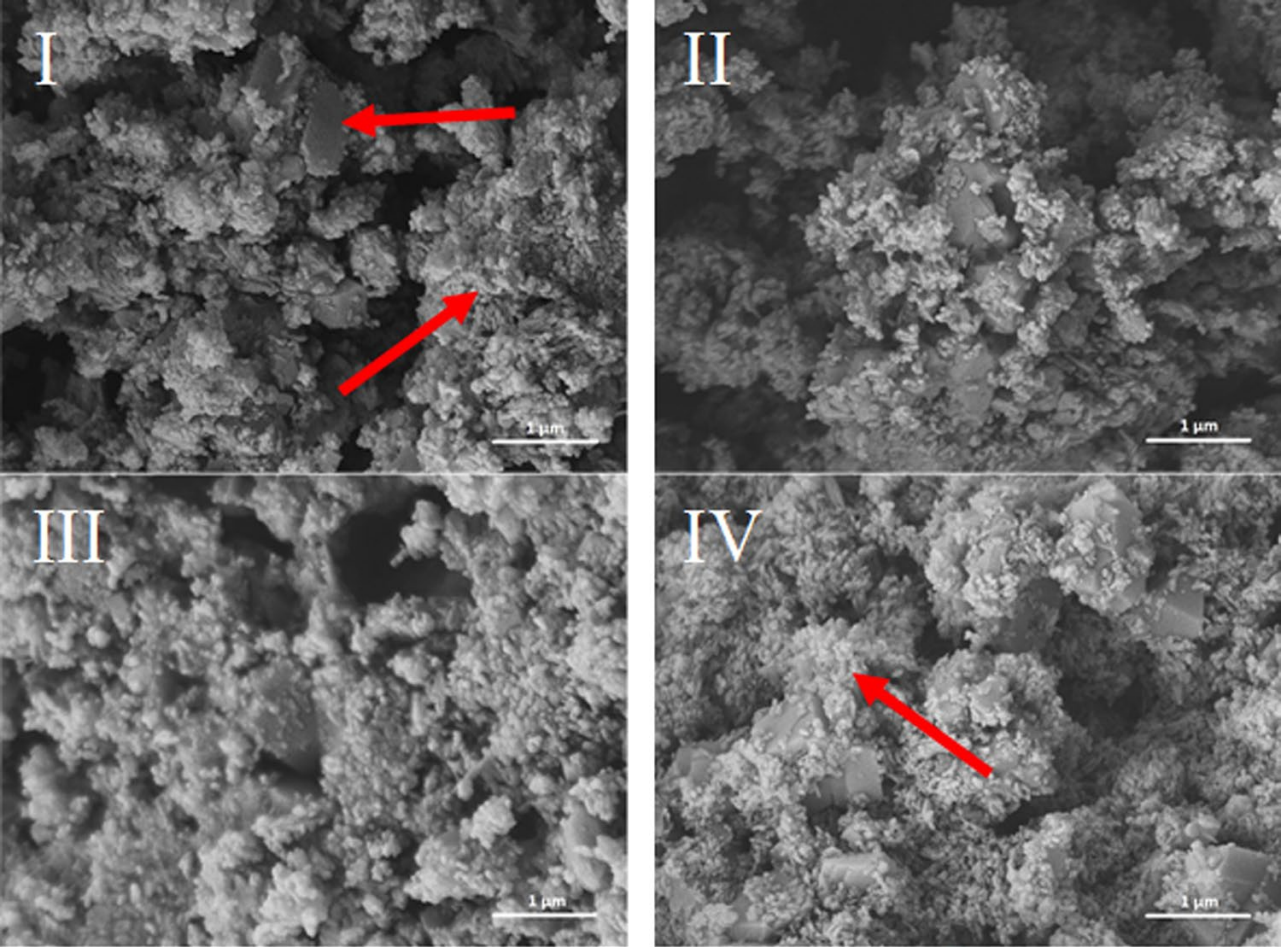
Lime putty SL-C at the age of 3 days (**I**), 30 days (**II**), 60 days (**III**) and 90 days (**IV**).

**Figure 6:**
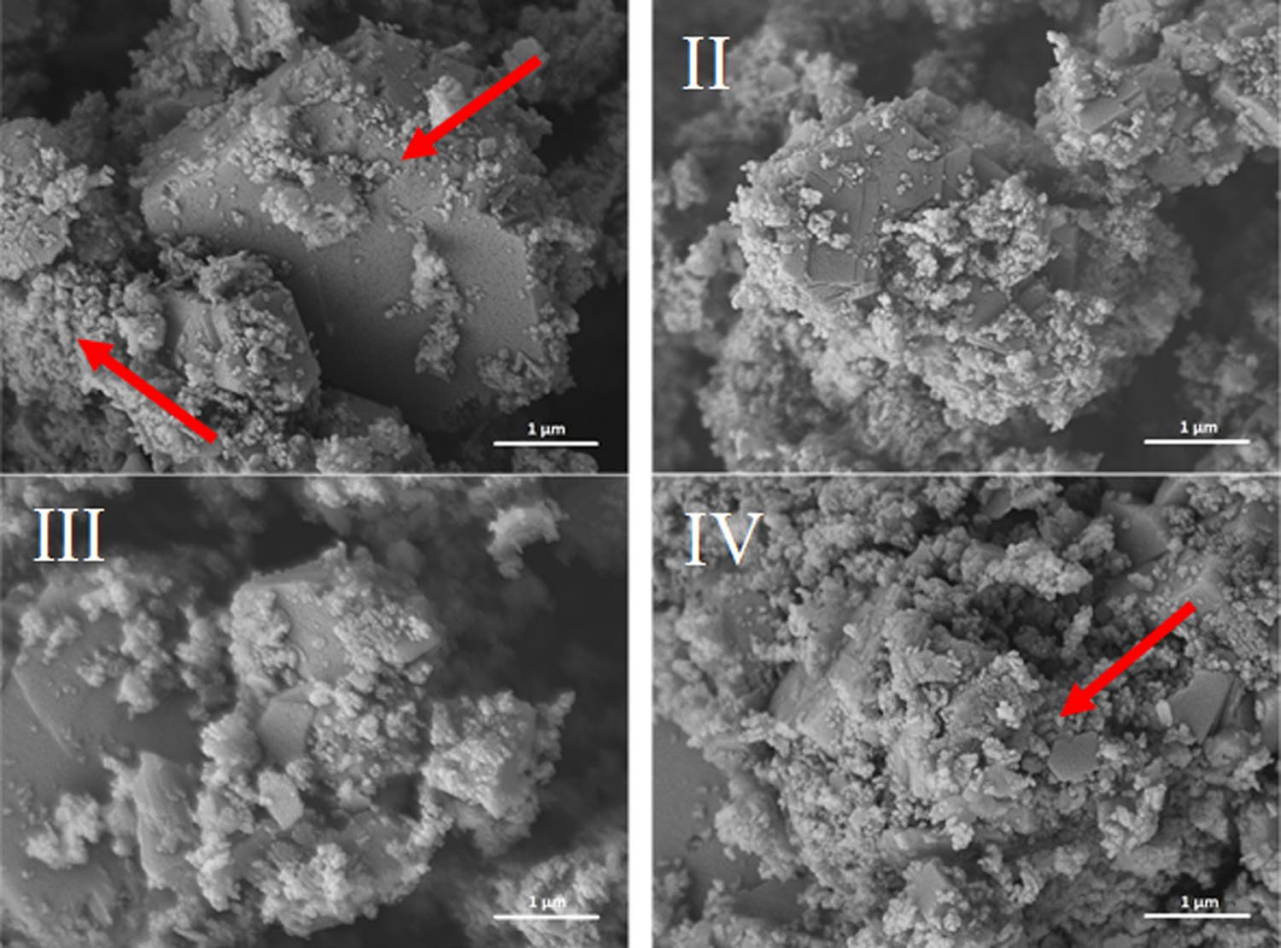
Lime putty SL-B1 at the age of 3 days (**I**), 30 days (**II**), 60 days (**III**) and 90 days (**IV**).

Figures 3–6 illustrate the microstructure of lime putties SL-A, SL-B, SL-C, and SL-B1 during their maturation at the age of 3, 30, 60, and 90 days. Figures 3I and 4I show the prismatic hexagonal crystals of calcium hydroxide created in samples SL-A and SL-B. Lime putties SL-C and SL-B1 do not contain the significant crystals of calcium hydroxide which are present in samples SL-A and SL-B. The crystals in these cases are coated with a layer of hydrogel (Ca(OH)_2_.aq), which is already discernible after three days of hydration (maturation) Figs 5I and 6I. During the maturation of all the lime putty samples, the regular hexagonal crystals of calcium hydroxide are transformed into smaller irregular crystals which are coated with a layer of hydrogel. The gradual transformation of the calcium hydroxide crystals is well evident in Figs 3II–IV, 4II–IV, 5II–IV, and 6II–IV. The transformation of the calcium hydroxide crystals and hydrogel formation of lime putties SL-A and SL-B is slower than in the samples SL-C and SL-B1. The micrographs in Figs 5III and 6III show the calcium hydroxide crystals coated with a layer of hydrogel which originates faster and more intensively in samples SL-C and SL-B1. Conversely, the micrographs in Figs 3III and 4III present the microstructure of lime putties SL-A and SL-B with clearly visible crystals of calcium hydroxide. The layer of hydrogel increases during the maturation of lime putties. The amount of hydrogel is greatest at the age of 90 days in sample SL-C and in sample SL-B1 Figs 5IV and 6IV. The formation of hydrogel on the surface of the crystals of calcium hydroxide proceeds best in sample SL-C, which was prepared from lump lime where the regular hexagonal crystals of calcium hydroxide were not fully developed. The process of particle disruption immediately after hydration enables the acceleration of the process of hydrogel formation on the surface of the crystals of calcium hydroxide, and, moreover, the process of transformation of the regular crystals of calcium hydroxide to the smaller irregular ones is faster. The microstructure images of lime putties confirm the results provided by the performed rheological measurements.

**Figure 7:**
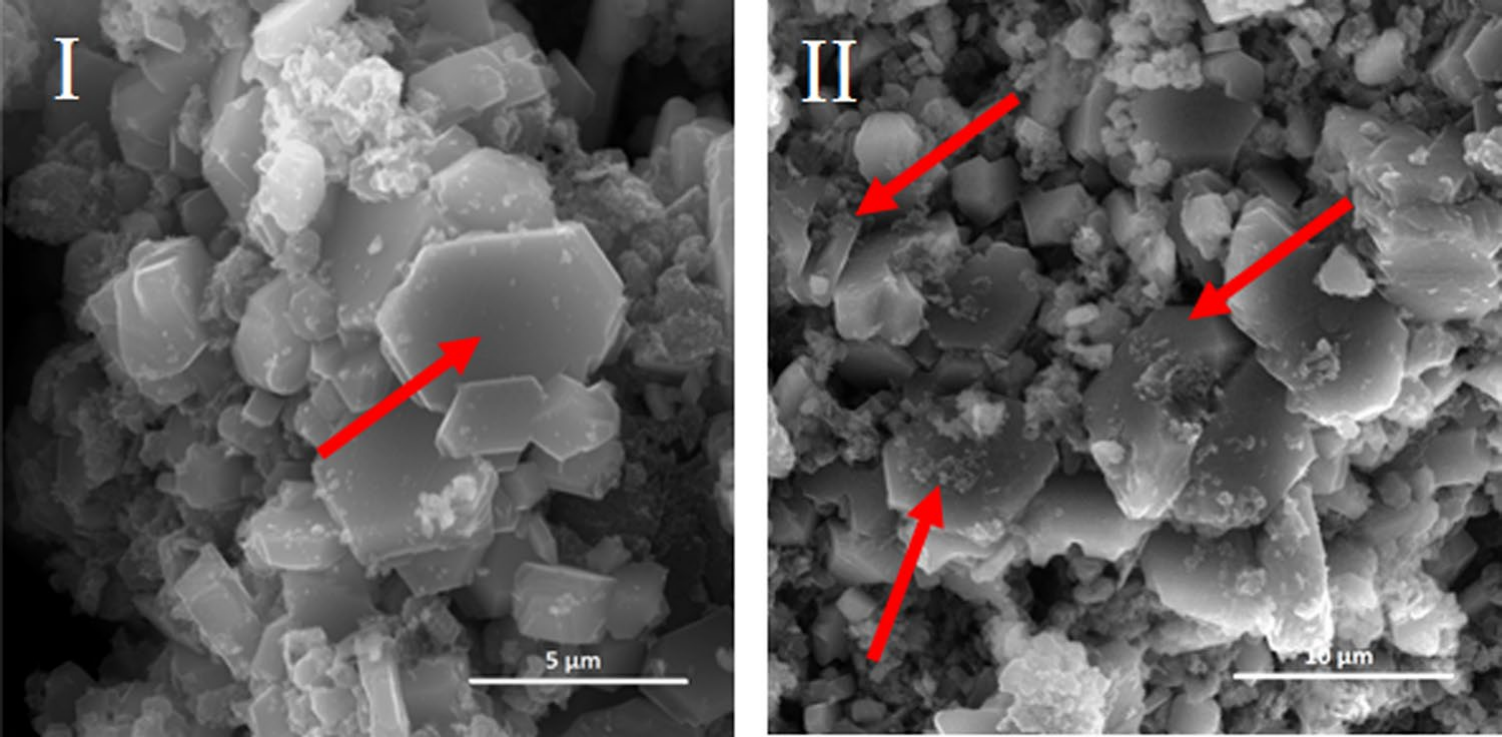
The lime putty SL-A at the age of 3 days (**I**) and 30 days (**II**).

The HR-ESEM images of lime putty SL-A without preparation of the sample and sputtering at the age of 3 and 30 days are shown in Fig. 7. Owing to the sophisticated and, in this work, experimentally proven method for the preparation of samples for HR-SEM, the images from the HR ESEM provide very similar information: significant hexagonal crystals of calcium hydroxide see Fig. 7I. The regular hexagonal crystals of calcium hydroxide are gradually transformed into smaller irregular crystals coated with a layer of hydrogel during the maturation of lime putty Fig. 7II. The HR-ESEM is a preferable method for observing micro-morphological changes during the maturation of lime putties because it is not necessary to use complicated sample preparation methods. Incorrectly sputter coated samples can lead to the formation of artefacts which can distort the micro-morphological interpretation. The HR-SEM is a suitable method for the evaluation of morphological changes during the maturation of lime putties; however, this method places heightened demands on the sample preparation, and, therefore, it can introduce inaccuracies into the characterization of the microstructure. The HR-ESEM is a more accurate, faster, and easier method for the evaluation of the morphology lime putties, as the samples are not treated at all before their observation^20,21^. Thus, there are no artefacts distorting the microstructural interpretation.

The same specific differences in the sample morphology may also be caused due to the samples drying and coating of a metal layer for SEM observation. Conversely, observation in ESEM does not require the above processes and the samples are observed naturally wet and without any modifications. The surface morphology of the crystals observed using the ESEM are somewhat smooth and free of sharp edges and micro-cracks see Fig. 7I and II vs. Fig. 3I and II.

### Microstructure of carbonated lime putties

**Figure 8:**
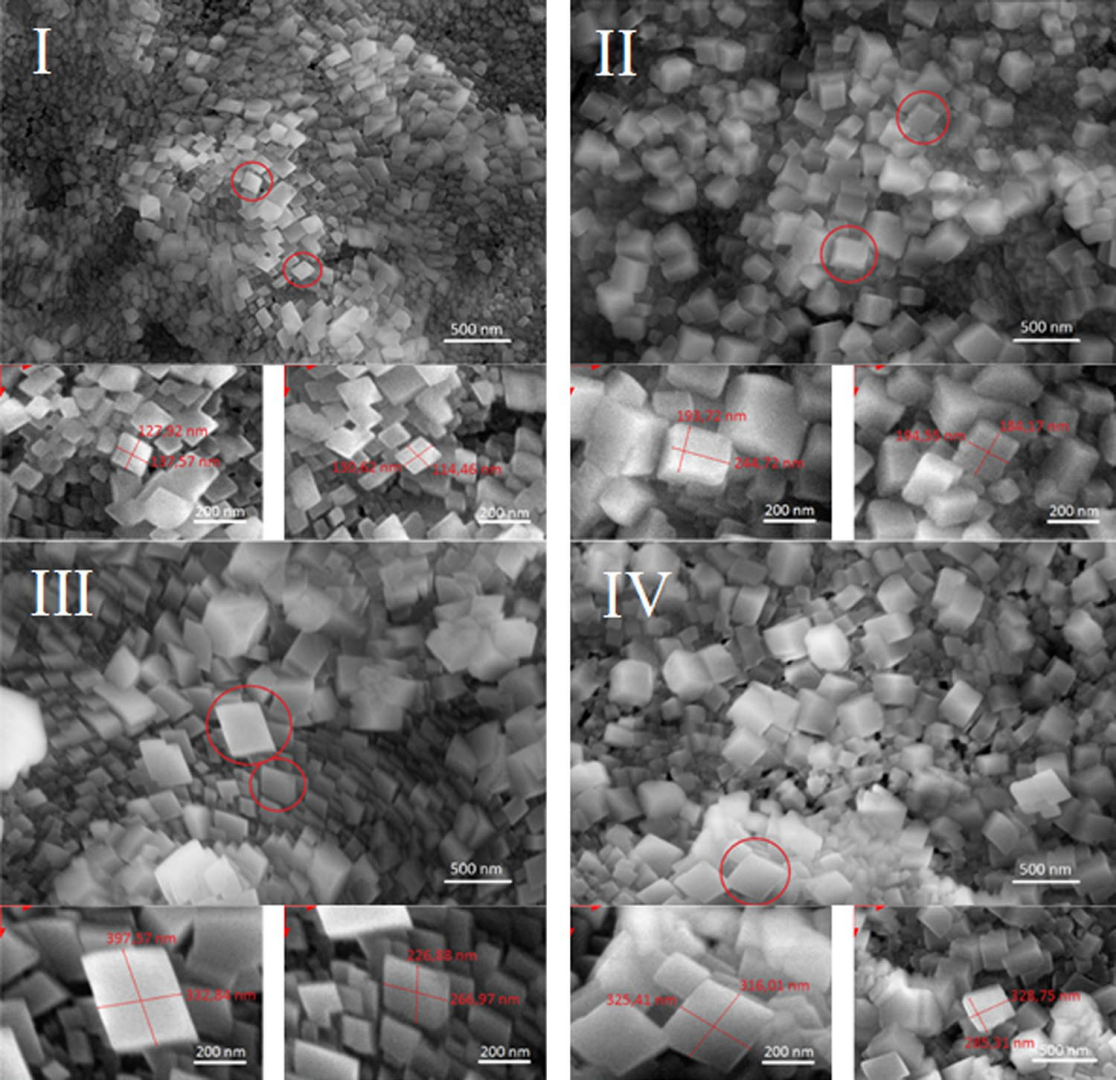
Microstructure of carbonated lime putties C-SL-A (**I**), C-SL-B (**II**), C-SL-C (**III**) and C-SL-B1 (**IV**).

Figure 8 shows the development of calcite crystals in carbonated lime putties C-SL-A, C-SL-B, C-SL-C, and C-CL-B1 after 14 days of storage in a box with a high concentration (20%) of CO_2_ and humidity (70%). The length of the side of the calcite crystals was measured. The regular crystals which grew separately were selected for measurement. The images in Fig. 8 and Table 1 show the different development of calcite crystals in the carbonated lime putties. The size of calcite crystals is affected by the preparation method of the lime putties and the granulometry of lime. The smallest calcite crystals were developed in lime putty C-SL-A, which was prepared from lime with a grain size of under 90 μm Fig. 8I; whereas the larger crystals originated in lime putty C-SL-B Fig. 8II. The largest calcite crystals were found in lime putty C-SL-C Fig. 8III. The method of preparation, i. e. the mixing of lime putty SL-B1, affected the size of the calcite crystals C-SL-B1 Fig. 8IV. The mixing process leads on to the easier dissolution of the grains of quicklime, subsequently to the formation of larger crystals of calcium hydroxide.

**Table 1:** Average side length of the calcite crystals. The repeatability limit was calculated for each value.

	C-SL-A	C-SL-B	C-SL-C	C-SL-B1
Average side length of calcite crystals side [nm]	128.84 ± 10.77	236.00 ± 13.18	309.67 ± 14.96	244.06 ± 14.18

### Differential thermal analysis of the carbonated lime putties

**Figure 9:**
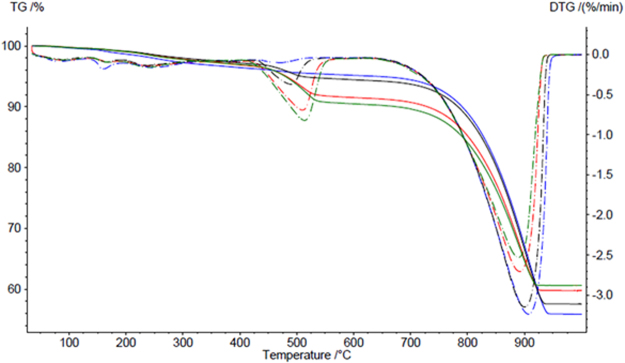
Differential thermal analysis of the carbonated lime putties C-SL-A, C-SL-B, C-SL-C and C-SL-B1, red curve – C-SL-A, green curve – C-SL-B, blue curve – C-SL-C, black curve – C-SL-B1.

The results of differential thermal analysis of carbonated lime putties C-SL-A, C-SL-B, C-SL-C, and C-CL-B1 after 14 days of storage in a CO_2_ environment are demonstrated in Fig. 9. The curves TG and DTG show that the carbonation process for the individual lime putties is different. The carbonation of lime putty samples C-SL-A and C-SL-B proceeded similarly. The smaller grains in lime A lead to a lower content of calcium hydroxide and a higher content of calcium carbonate (25.2% Ca(OH)_2_ and 65.7% CaCO_3_) compared to lime B (20.0% Ca(OH)_2_ and 68.7% CaCO_3_). Sample C-SL-C, prepared from lump lime, carbonates very quickly (2.8% Ca(OH)_2_ and 85.8% CaCO_3_). Sample C-SL-B1 contained less calcium hydroxide and, actually, more calcium carbonate (8.9% Ca(OH)_2_ and 80.0% CaCO_3_) than lime putty C-SL-B due to the process of activation.

### Strengths of lime mortars

The effect of lime putty preparation on the carbonation process was also assessed by determining the strengths of lime mortars. The results of the tensile and compressive strengths measurements of lime mortars M-A, M-B, M-C, and M-B1 at the age of 28 days are illustrated in Table 2. The strengths of the lime mortars vary depending on the granulometry of the original quicklime from which the lime putties and the mortars were prepared. Mortar M-B, prepared from lime putty SL-B, reached higher strengths than mortar M-A, which was prepared from lime putty SL-A. Mortar M-C, which was prepared from the putty made from lump lime, reached the highest strengths.

**Table 2:** Average strengths of lime mortars M-A, M-B, M-C and M-B1 at 28 days. The repeatability limit was calculated for each value.

	MA	MB	MC	MB1
Flexural strength [MPa]	0.5 ± 0.01	0.6 ± 0.02	0.8 ± 0.02	0.7 ± 0.03
Compressive strength [MPa]	1.3 ± 0.06	1.5 ± 0.05	2.5 ± 0.08	1.8 ± 0.05

The effect of the method of preparation of lime putty is also noticeable because mortar M-B1 achieved higher strengths than mortar M-B. Both of these lime mortars were prepared from lime with the same granulometry, but lime putty SL-B1 was mixed, unlike lime putty SL-B.

## Discussion

The aim of the article was to analyse the development of the lime putties in the dependence on time by means of selected methods giving accurate results. The found results exactly characterize the lime putties’ properties and correspond with the results published by other authors. The authors of the article consider a high potential in the micro-morphological characterization of the lime putties in their native state, so without any sample treatment with minimum occurrence of artefacts, by means of ESEM. The article presents an evaluation of the results obtained by means of classic SEM and ESEM for the objective arbitration.

Four lime putties prepared in different ways from quicklimes with varying granulometry were analysed in the dependence on the maturation time. During the maturation of lime putties, the regular hexagonal crystals of calcium hydroxide are transformed into smaller irregular crystals which are coated with a layer of hydrogel. The rheological properties are dependent on the amount of hydrogel produced during the ageing of lime putties.

- The use of lump lime or mixing a suspension of lime for the preparation of lime putty leads to the formation of incompletely regular calcium hydroxide crystals and a high amount of hydrogel formation. It results in a faster formation of calcium carbonate, the growth of larger calcite crystals, and higher strengths of lime mortars.
- The values of plastic viscosity and yield stress increase with the increasing period of maturation. The use of aged limes leads to obtaining an intermediate product of higher quality for the preparation of lime mortars.
- The process of particle disruption during the mixing of lime putty helps accelerate the hydrogel formation as well as increasing plastic viscosity and yield stress.
- The use of lump lime or the mechanical disruption of lime particles is the most suitable for the properties of lime putty and, consequently, for the preparation of lime mortars.
- Matured lime putties prepared from lump lime and by mixing lime putty are the most suitable for the preparation of mortar.

The most important contribution of the article in the field of conservation and restoration of historical mortars consists in the new way of lime putties preparation by means of mechanical crush of the lime particles. Mortars produced from such prepared lime putty achieved similar properties as mortars made from the matured lime putty prepared from lump lime. An advantage of the new way of lime putty preparation is the use of commonly available powder lime without the necessity of using more difficult to obtain lump lime. The presented results exhibited that mortars with higher strengths can be prepared from common powder lime. These mortars will be more durable with a higher resistance to environmental influences and contaminated atmosphere.

## Methods

### Limes

Lime A with a grain size of under 90 µm, lime B with a grain size of under 200 µm, and lump lime with a grain size from 10 mm to 63 mm (Lime kiln, Čertovy schody a.s., Tmaň) were used to prepare the lime putties. Lime A and lime B are mixtures of lime burned partially (70%) in the co-current regenerative furnace at 1000 °C and in the countercurrent shaft furnace (30%) at 1300 °C. Lime C is a mixture of lime burned in the co-current regenerative furnace at 1100 °C (50%) and in the countercurrent shaft furnace at 1300 °C (50%). The content of total CaO/active CaO in lime A was 96.05/93.74, in lime B 94.08/88.34, and in lime C 95.87/92.33. The reactivity of lime expressed as the time taken to reach a temperature of 60 °C, according to EN 459-2, was 2.3 min for lime A, 2.6 min for lime B, and 3.5 min for lime C.

The granulometry of lime A and lime B is shown in Table 3. Lime A contains more fine particles under 0.063 µm and no particles above 200 µm. Lime B contains less fine particles under 0.063 µm and exhibits a higher content of particles below 200 µm and above 200 µm.

**Table 3:** Grain content of lime A and B.

Lime	<0.063 [µm]	0.063–0.090 [µm]	0.090–0.200 [µm]	>200 [µm]
A	15.18	14.04	3.41	2.77
B	14.14	9.85	4.62	2.86

### Preparation of the lime putties and the lime mortars

Lime putties SL-A, SL-B, and SL-C were prepared by mixing 200 g of lime and 640 ml of deionized water. Lime putty SL-B1 was prepared by mixing 200 g of lime and 640 ml of deionized water and this lime putty was mixed (25,000 rev/min) for 5 minutes after its hydration. The fresh lime putties were immediately stored in plastic bottles and they were supplemented with water to avoid reaction with CO_2_ and air drying. The samples were then kept in closed bottles at a temperature of 20 ± 1 °C. Samples SL-A, SL-B, SL-C, and SL-B1 were stored in a box with 70% humidity and a 20% concentration of CO_2_ for 14 days for accelerated carbonation. Lime mortars M-A, M-B, M-C, and M-B1 were prepared from the lime putties and sand with a ratio of 1:3. The lime mortars were placed in moulds with dimensions of 40 × 40 × 160 mm. Specimens were stored under laboratory conditions at a temperature of 20 ± 1 °C and a relative humidity of 50 ± 5% after their removal from the moulds.

### Rheology

The rheological properties of lime putties SL-A, SL-B, SL-C, and SL-B1after 3, 30, 60, and 90 days’ maturation were monitored by a Discovery Hybrid Rheometer HR-1 (parallel plate diameter of 25 mm with roughened surface). The rheological properties of the lime putties were determined from the curves of flow with the increasing and decreasing shear rate in the range from 0 sec^−1^ to 200 sec^−1^ over 2 minutes. The values of yield stress and plastic viscosity were determined according to the Bingham model,

2 $$\tau ={\tau }_{0}+\mu \dot{\gamma }$$

where *τ* [Pa] is the shear stress,*τ*_0_ is the yield stress, *μ* [Pa·s] is the plastic viscosity, and $\dot{\gamma }$ [s^−1^] is the shear strain rate^22^. Temperature of the measurement apparatus and the laboratory were kept constant at 20 (±0.5) °C.

### Observation in SEM and ESEM

Lime putty samples SL-A, SL-B, SL-C, and SL-B1 were observed in the high resolution scanning electron microscope (HR-SEM) JSM6700F JEOL (with a beam energy of 5 keV, a working distance of 8 mm, and an Everhart-Thornley detector) after 3, 30, 60, and 90 days. The samples of the lime putties were prepared for observation in the SEM in the following manner: the samples were rinsed with 10 ml of acetone to remove water and 10 ml of diethyl ether for drying. Next, the samples were dried in a nitrogen atmosphere in order to eliminate the influence of CO_2_, and the samples were sputtered.

The sample of lime putty SL-A was observed in the environmental scanning electron microscope (ESEM) AQUASEM II (with a beam energy of 20 keV, a water vapour pressure of 800 Pa, and an ionization detector) after 3 and 30 days. The sample was studied directly without sputtering.

The different development of the calcite crystals in carbonated lime putties C-SL-A, C-SL-B, C-SL-C a C-SL- B1 was monitored with the high resolution environmental scanning electron microscope (HR-ESEM) QUANTA 650 FEG (with a beam energy of 10 keV, water vapour pressure of 200 Pa, and a GSED detector). The samples were studied directly without sputtering. The length of the side of the calcite crystals was measured using the Scandium software.

### Differential thermal analysis

The differential thermal analysis of carbonated lime putties C-SL-A, C-SL-B, C-SL-C, and C-SL-B1 was performed using the thermal analyser NETSCH STA 2500 after 14 days of being stored in a box with 70% humidity and a 20% concentration of CO_2_. The used instrument exhibits accuracy with an error ±10%. The measurement was carried out under a nitrogen atmosphere.

### Strengths

The flexural strength and compressive strength of lime mortars M-A, M-B, M-C, and M-B1 were determined at 28 days according to EN 1015-11.

All presented data was calculated as average value from the repeated five measurements and standard deviation and repeatability limit (probability level 95%) were calculated.
